# Incremental Value of Myocardial Work over Global Longitudinal Strain in the Surveillance for Cancer-Treatment-Related Cardiac Dysfunction: A Case–Control Study

**DOI:** 10.3390/jcm11040912

**Published:** 2022-02-09

**Authors:** Wojciech Kosmala, Tomoko Negishi, Paaladinesh Thavendiranathan, Martin Penicka, Jonathan De Blois, Klaus Murbræch, Sakiko Miyazaki, Mitra Shirazi, Ciro Santoro, Dragos Vinereanu, Goo-Yeong Cho, Krassimira Hristova, Bogdan A. Popescu, Koji Kurosawa, Masaki Izumo, Kazuaki Negishi, Monika Przewlocka-Kosmala, Thomas H. Marwick

**Affiliations:** 1 Institute of Heart Diseases, Wroclaw Medical University, 50-345 Wroclaw, Poland; monika.przewlocka-kosmala@umw.edu.pl; 2 Baker Heart & Diabetes Institute, Melbourne, VIC 3004, Australia; Tom.Marwick@baker.edu.au; 3 Menzies Institute for Medical Research, University of Tasmania, Hobart, TAS 7000, Australia; tomoko.negishi@utas.edu.au (T.N.); kazuaki.negishi@sydney.edu.au (K.N.); 4 Sydney Medical School Nepean, Charles Perkins Centre Nepean, Faculty of Medicine and Health, The University of Sydney, Sydney, NSW 2000, Australia; 5 Ted Rogers Program in Cardiotoxicity Prevention, Peter Munk Cardiac Center, Toronto General Hospital, University of Toronto, Toronto, ON M5S 1A1, Canada; dinesh.thavendiranathan@utoronto.ca; 6 Cardiovascular Center Aalst, 9300 Aalst, Belgium; martin.penicka@olvz-aalst.be; 7 Centre de Recherche du CHU de Québec, Hôpital Saint-Sacrement, Québec City, QC G1S4L8, Canada; Jonathan.Deblois@fmed.ulaval.ca; 8 Oslo University Hospital, N-0424 Oslo, Norway; sbmurk@ous-hf.no; 9 Juntendo University Hospital, Tokyo 113-8421, Japan; smiyaza@juntendo.ac.jp; 10 Royal Adelaide Hospital, Adelaide, SA 5000, Australia; mgh_shirazi@yahoo.com; 11 Department of Translational Medical Sciences, Federico II University of Naples, 80138 Naples, Italy; cirohsantoro@gmail.com; 12 Emergency Institute for Cardiovascular Diseases, Bucharest University Emergency Hospital, University of Medicine and Pharmacy Carol Davila, 020021 Bucharest, Romania; vinereanu@gmail.com (D.V.); bogdan.a.popescu@gmail.com (B.A.P.); 13 Seoul National University Bundang Hospital, Seongnam-si 13620, Korea; cardioch@snu.ac.kr; 14 National Heart Hospital, 1309 Sofia, Bulgaria; khristovabg@yahoo.com; 15 Department of Medicine and Biological Science, Gunma University, Maebashi 371-8511, Japan; chachamaru49414@yahoo.co.jp; 16 Department of Cardiology, St. Marianna University School of Medicine, Kawasaki 216-8512, Japan; heartizumo@yahoo.co.jp

**Keywords:** chemotherapy, cardiotoxicity, myocardial work, myocardial strain, left ventricular longitudinal deformation

## Abstract

The load dependence of global longitudinal strain (GLS) means that changes in systolic blood pressure (BP) between visits may confound the diagnosis of cancer-treatment-related cardiac dysfunction (CTRCD). We sought to determine whether the estimation of myocardial work, which incorporates SBP, could overcome this limitation. In this case–control study, 44 asymptomatic patients at risk of CTRCD underwent echocardiography at baseline and after oncologic treatment. CTRCD was defined on the basis of the change in the ejection fraction. Those with CTRCD were divided into subsets with and without a follow-up SBP increment >20 mmHg (CTRCD+BP+ and CTRCD+BP−), and matched with patients without CTRCD (CTRCD−BP+ and CTRCD−BP−). The work index (GWI), constructive work (GCW), wasted work (GWW), and work efficiency (GWE) were assessed in addition to the GLS. The largest increases in the GWI and GCW at follow-up were found in CTRCD−BP+ patients. The CTRCD+BP− patients demonstrated significantly larger decreases in GWI and GCW than their CTRCD+BP+ and CTRCD−BP− peers. ROC analysis for the discrimination of LV functional changes in response to increased afterload in the absence of cardiotoxicity revealed higher AUCs for GCW (AUC = 0.97) and GWI (AUC = 0.93) than GLS (AUC = 0.73), GWW (AUC = 0.51), or GWE (AUC = 0.63, all *p*-values < 0.001). GCW (OR: 1.021; 95% CI: 1.001–1.042; *p* < 0.04) was the only feature independently associated with CTRCD−BP+. Myocardial work is superior to GLS in the serial assessments in patients receiving cardiotoxic chemotherapy. The impairment of GLS in the presence of an increase in GWI and GCW indicates the impact of elevated afterload on LV performance in the absence of actual myocardial impairment.

## 1. Introduction

Cancer-related mortality has declined substantially over recent decades [1]. However, improved survival has led the cardiac toxicity of anti-cancer treatment to become a major cause of decreased quality of life and, in some cases, premature death, among cancer survivors [2,3,4]. In particular, anthracycline cardiomyopathy confers an especially poor prognosis, with a 2-year mortality reaching 60% [5]. Accordingly, the early detection and management of cancer-treatment-related cardiac dysfunction (CTRCD) should be prioritized in patients receiving potentially cardiotoxic chemotherapy.

Current guidelines primarily recommend the left ventricular ejection fraction (LVEF) to monitor LV systolic function and to identify cardiotoxicity [4,6]. Observational studies indicating global longitudinal deformation (GLS) to be superior to EF in the recognition of subclinical myocardial impairment and prediction of outcome after exposure to cancer therapy [7,8,9,10,11,12] have recently been confirmed by the 12-month results of the SUCCOUR trial [13]. However, all the ejection-phase parameters are afterload-dependent and vulnerable to fluctuations in blood pressure, which poses a particular problem with serial assessments. The estimation of myocardial work (MW), to allow the correction of GLS for changes in systolic blood pressure [14,15,16,17], may improve diagnostic accuracy in this setting. Thus, in this study, we sought to investigate the utility of MW parameters in the differentiation between the actual chemotherapy-related and afterload elevation-related alterations in GLS.

## 2. Materials and Methods

**Study subjects.** The population of this case–control study was derived from participants in the prospective randomized SUCCOUR trial, which assessed the usefulness of GLS in the guidance of cardioprotective treatment in at-risk patients undergoing potentially cardiotoxic chemotherapy [18]. We included 44 subjects (mean age: 51 ± 9 years; 91% female) receiving anthracyclines in combination with one or more of three additional risks. The first was the presence of current (but not concurrent) trastuzumab in breast cancer with the HER2 mutation or tyrosine-kinase inhibitors (e.g., sunitinib). The second was the presence of a cumulative anthracycline dose >450 g/m^2^ of doxorubicin (or equivalent cumulative dose of another anthracycline, e.g., epirubicin > 900 g/m^2^). The third was an increased risk of heart failure (any two of age > 65 years, type 2 diabetes mellitus, hypertension, and previous cardiac injury, e.g., myocardial infarction). We excluded patients with heart failure or LV dysfunction at baseline (EF < 50%), valvular stenosis or regurgitation of more than moderate severity, a history of previous heart failure (baseline NYHA > 2), systolic BP < 110 mmHg, a heart rate <60/min, an inability to acquire interpretable echocardiographic images, contraindications for/intolerance to beta-blockers or ACE inhibitors, existing therapy with both beta-blockers and ACE inhibitors, and an oncologic (or other) life expectancy < 12 months.

Echocardiographic assessment was performed at baseline and repeated on a 3-monthly basis during the first year after recruitment. CTRCD was defined by a 10% asymptomatic fall of LVEF to <55% [19]. To test the hypothesis that myocardial work was superior to GLS, we designated four groups, based on changes in LV systolic function and blood pressure relative to baseline measurements:

i. CTRCD+BP−: cardiotoxicity and no increase in afterload (defined arbitrarily as an increase in SBP > 20 mmHg;) at a follow-up examination;
ii. CTRCD+BP+: cardiotoxicity with a concomitant increase in afterload at a follow-up examination;
iii. CTRCD−BP+: no cardiotoxicity in the presence of an increase in afterload at a follow-up examination;
iv. CTRCD−BP−: no cardiotoxicity and no increase in afterload at a follow-up examination.

“Follow-up examination” denotes echocardiography at the time of cardiotoxicity recognition and/or SBP elevation during examination (groups i–iii), or at the routine follow-up echocardiographic study in group iv.

Based on 11 individuals eligible for the CTRCD+BP+ group, we used individual matching based on sex and age (±6 years), forming 11 compatible quadruplets.

The SUCCOUR trial was designed in accordance with the Declaration of Helsinki, and was approved by the institutional review boards of the participating institutions. The patients were informed of the purpose of the study and provided written informed consent. This work represents an unplanned substudy of the trial, using site measurements of GLS and EF, independent of randomization to strain-guided or usual care.

**Echocardiography.** Echocardiographic imaging was carried out using Vivid E9 and Vivid E95 equipment (GE Medical Systems, Horten, Norway) with phased-array 2.5 MHz multifrequency transducers. Multiple consecutive cardiac cycles of standard echocardiographic views were acquired and stored digitally for subsequent analysis. Each baseline and follow-up assessment of LV function was performed by a single expert observer who was blinded to the study data.

*Conventional and tissue Doppler imaging*. The measurements of cardiac dimensions and wall thicknesses were performed according to the recommendations of the American Society of Echocardiography [20]. The LV ejection fraction was calculated by real-time 3-dimensional echocardiography as previously described [21]. The mitral inflow and mitral annular indices were measured using the apical four-chamber view. The ratio of the mitral inflow early diastolic velocity to the average early diastolic tissue velocity obtained from the septal and lateral sides of the mitral annulus (E/e’) was calculated to approximate the LV filling pressure.

*Speckle tracking imaging*. Longitudinal myocardial deformation was assessed using semi-automated 2D speckle tracking (Echopac PC version 202, GE Medical Systems) in the three apical views with typical temporal resolutions of 50–70 frames/s. The timing of the aortic and mitral valve opening and closure was obtained using pulsed-wave Doppler traces. After the manual tracing of the endocardial border and selecting the appropriate wall thickness, the software automatically identified 6 segments in each view and tracked the motion of acoustic markers. Segments with inadequate tracking were readjusted manually, and, if this was unsuccessful, they were excluded from further analysis. After measuring the greatest negative value on the strain curve, GLS was calculated as the average from all the LV segments interrogated. All the echocardiographic parameters were averaged over 3 consecutive cardiac cycles.

*Myocardial work evaluation*. Myocardial work was assessed by the combination of LV strain data and a non-invasively estimated LV pressure curve, as previously described [22]. Based on the assumption that the peak LV systolic pressure was equal to the SBP measured with a cuff manometer, the patient-specific LV pressure curve was constructed by adjusting the standard LV pressure curve to the duration of the isovolumic and ejection phases defined by the timing of valvular events. Strain and pressure data were synchronized using the onset of the R-wave in the ECG, and the area of the pressure strain loop served as an index of myocardial work (WI). Constructive work (CW) was defined as the work during segmental shortening in systole and during lengthening in isovolumic relaxation. Conversely, the myocardial work performed during lengthening in systole and shortening in isovolumic relaxation, associated with energy loss, was termed wasted work (WW). Constructive work divided by the sum of constructive and wasted work represented the myocardial work efficiency (WE). Global values were obtained as averages from all the segmental values of the MW parameters, including the global myocardial work index (GWI), global constructive work (GCW), global work waste (GWW), and global work efficiency (GWE).

Blood pressure was measured in the supine position immediately before the echocardiographic study after a 5 min rest using an appropriately sized cuff according to the arm circumference.

**Statistical analysis.** Data are presented as means ± SDs Between-group comparisons were performed using analysis of variance (ANOVA), with the Scheffe post hoc test for continuous variables and the chi-square test for categorical variables. The homogeneity of the variances was evaluated using the Levene test. Logistic regression analysis was used to assess the associations of the GLS and MW indices with the LV response to increased afterload. A receiver operator characteristic (ROC) analysis was used to examine the ability of the GLS and MW indices to identify the LV response to increased afterload. Differences in the area under the ROC curves (AUCs) were analyzed using the z-test. Changes in particular parameters during follow-up were calculated by subtracting the baseline value from the follow-up value and were expressed in the units of their measurements. All the analyses were performed with standard statistical software (Statistica version 13, TIBCO Software Inc., Palo Alto, CA, USA). The level of statistical significance was set at a two-sided *p*-value < 0.05.

## 3. Results

**Participant characteristics.** The oncologic diagnosis was breast cancer in 40 and hematologic malignancies in four patients. The chemotherapy included two different drug regimens—one based exclusively on anthracyclines, and the other, on a combination of anthracyclines and trastuzumab.

CTRCD was detected in seven patients at 3-month follow-up, in eight at 6 months, in five at 9 months, and in two at 12 months. The demographic, clinical, and echocardiographic characteristics of the subsets separated according to the development of cardiotoxicity and increase in blood pressure at follow-up are presented in Table 1 and Table 2. No significant intergroup differences were shown.

**Changes in metrics of LV function at follow-up.** A significantly greater decrease in GLS at follow-up was noted in the CTRCD+BP+ group as compared to the subsets without cardiotoxicity. Larger increases in the GWI and GCW were found in CTRCD−BP+ than in the other three groups. Patients from the CTRCD+BP− group demonstrated significantly larger decreases in the GWI and GCW than their peers from the CTRCD+BP+ and CTRCD−BP− groups. The other parameters of myocardial work—the GWW and GWE—did not differ among the subsets. By definition, the decrease in the LV ejection fraction at follow-up was significantly greater in both groups with cardiotoxicity (Table 3).

**Figure 1:**
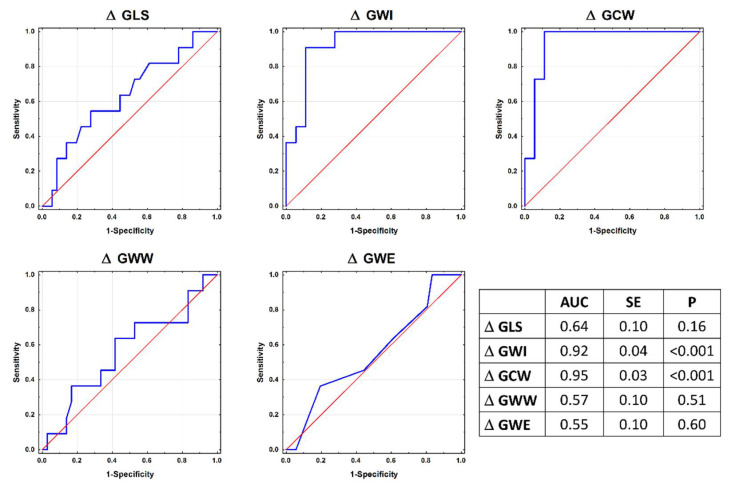
ROC curves defining the usefulness of global longitudinal deformation and myocardial work parameters (changes from baseline to follow-up) for identification of LV response to increased afterload with no myocardial impairment. *p*-values for differences: GLS vs. GWI, <0.001; GLS vs. GCW, <0.001; GLS vs. GWW, 0.16; GLS vs. GWE, 0.27; GWI vs. GCW, 0.19; GWI vs. GWW, <0.001; GWI vs. GWE, <0.001; GCW vs. GWW, <0.001; GCW vs. GWE, <0.001; GWW vs. GWE, 0.51.

**Identification of LV response to increased afterload with no myocardial impairment.** Changes in the GWI and GCW at follow-up were more useful for the identification of LV functional changes in response to increased afterload without satisfying the arbitrary criteria for cardiotoxicity (defined as CTRCD−BP+ group membership) than changes in the GLS, GWW, and GWE (all *p*-values < 0.001; Figure 1). In a series of bi-variable logistic regression models, each encompassing the GLS and myocardial work parameter, the change in GCW at follow-up was the only significant correlate of CTRCD (OR: 1.021; 95% CI: 1.001–1.042; *p* < 0.04).

**Reproducibility.** The intraobserver variability of measurements assessed in 15 randomly selected examinations and expressed as coefficients of variation was 5.2% for GLS, 5.3% for the GWI, 3.3% for GCW, 15.8% for GWW, and 1.7% for GWE.

## 4. Discussion

This study shows that the measurement of the MW might be helpful in the assessment of cardiotoxicity, especially when there is a BP change >20 mmHg. An increase in the GCW and GWI, even with decreased GLS, indicates the impact of elevated afterload on LV performance in the absence of actual myocardial impairment. From a practical point of view, the evaluation of MW may be a useful adjunct to GLS in patients with significant BP fluctuations during follow-up.

The strategy of the early detection of cardiotoxicity consists of serial echocardiographic imaging to identify LV dysfunction. The adopted definition of cardiotoxicity requires a 5% symptomatic or 10% asymptomatic reduction of EF to <55% [19]. However, an EF-based approach has several limitations including the technique-related variability of measurements and failure to detect early subtle changes [19,23,24]. The drop in EF may reflect advanced myocardial damage, unable to recover despite intervention in a substantial proportion of patients [25,26,27,28].

A growing body of research supports the incremental value of GLS over EF for monitoring the safety of chemotherapy [29]. In particular, the identification of LV dysfunction by this approach can be accomplished earlier than with conventional echocardiographic measures [8,10,11]. However, the diagnostic precision of LV-deformation assessment can be diminished by concomitant variations in blood pressure. The reason for this is the fact that the peak strain value depends on the joint effect of the contractile properties of heart muscle and the opposing force (afterload) during systole [14]. Thus, alterations in systolic blood pressure (both increments and decrements) may contribute to temporal changes in GLS from visit to visit and lead to a false conclusion on the myocardial status. The implementation of the MW, correcting the information obtained from LV deformation for LV pressure, facilitates the interpretation of myocardial contractility in relation to afterload.

In this study, we demonstrated a difference in the ability of MW and GLS in the recognition of blood-pressure-elevation-induced LV systolic function decline in the absence of the current definition of cardiotoxicity. This clinical scenario is recognized as a substantial increase in GCW and GWI, which is uncommon for cardiotoxicity. The lower decrease in the GWI and GCW in the CTRCD+BP+ patients as compared to the CTRCD+BP− subset may indicate some degree of LV contractility reserve in response to the elevation of LV afterload. Conceivably, a pattern of more profoundly decreased myocardial deformation and less-impaired GWI and GCW might be a marker of this group.

**Limitations.** Several study limitations should be acknowledged. The numbers of patients in each subcategory were small, and the case–control design might have provided a selection bias. Anthracyclines and trastuzumab—the two major cardiotoxic drugs used in this study—are characterized by different myocardial pathophysiology. However, the combined use of these medications in the majority of patients and sample-size limitations precluded the possibility of testing the utility of MW for each individual drug. We did not use biomarkers in this analysis; however, their usefulness for the detection of subclinical myocardial dysfunction (especially for trastuzumab-associated cardiomyopathy) is still controversial. Although a brachial cuff pressure was used to estimate the LV pressure in the calculation of the MW, which may not precisely reflect the hemodynamic profile during LV ejection, this method has been used in previous studies [22,30]. Finally, the limitation of imaging data analysis by a single observer might have led to an underestimation of the actual variability of the strain and myocardial work measurements.

**Clinical implications.** Our results have potential clinical implications for the cardioprotective management of patients undergoing oncologic treatment. The distinction between true cardiotoxicity secondary to chemotherapy and alterations in LV function resulting from afterload variations may be of prognostic significance. By providing a measure of myocardial performance independent of LV afterload, MW may be an important advance in the diagnostic workup in patients receiving anticancer treatment. The increasing role of GLS in the cardiac monitoring of chemotherapy might be complemented by the use of MW in some situations.

## Figures and Tables

**Table 1:** Baseline demographic and clinical characteristics in the studied population.

Parameter	CTRCD+ BP− *n* = 11 (A)	CTRCD+ BP+ *n* = 11 (B)	CTRCD−BP+ *n* = 11 (C)	CTRCD−BP− *n* = 11 (D)	P A−B	P A−C	P A−D	P B−C	P B−D	P C−D
Age, years	51.8 ± 10.3	48.4 ± 10.1	53.1 ± 7.6	50.4 ± 10.2	0.77	0.99	0.99	0.59	0.82	0.94
Body Mass Index, kg/m^2^	24.8 ± 3.7	26.8 ± 6.8	24.7 ± 2.4	27.5 ± 9.9	0.89	0.99	0.81	0.87	0.99	0.78
Hypertension, *n* (%)	2 (18)	3 (27)	0 (0)	3 (27)	0.62	0.15	0.62	0.06	1.00	0.06
Diabetes Mellitus, *n* (%)	0 (0)	3 (27)	2 (18)	0 (0)	0.06	0.15	1.00	0.62	0.06	0.15
Family History of Heart Failure, *n* (%)	4 (36)	2 (18)	3 (27)	3 (27)	0.35	0.65	0.65	0.62	0.62	1.00
Hemoglobin, g/dL	12.4 ± 1.3	11.4 ± 2.2	11.0 ± 1.6	12.1 ± 1.6	0.54	0.24	0.99	0.96	0.61	0.26
eGRF, ml/min/1.73 m^2^	95 ± 22	106 ± 29	104 ± 22	105 ± 22	0.85	0.93	0.91	0.99	0.99	0.99
Systolic Blood Pressure, mmHg	125 ± 15	115 ± 5	118 ± 13	127 ± 19	0.65	0.90	0.98	0.93	0.33	0.64
Diastolic Blood Pressure, mmHg	79 ± 10	76 ± 7	76 ± 10	77 ± 15	0.95	0.94	0.98	0.99	0.99	0.99
Medications										
Betablockers, *n* (%)	1 (9)	1 (9)	0(0)	0 (0)	1.00	0.32	0.32	0.32	0.32	1.00
ACEI/ARB, *n* (%)	1 (9)	1 (9)	0 (0)	0 (0)	1.00	0.32	0.32	0.32	0.32	1.00
Chemotherapy: A/A-T, *n*/*n* (%/%)	1/10 (9/91)	2/9 (18/82)	2/9 (18/82)	1/10 (9/91)	0.54	0.54	1.00	1.00	0.54	0.54

A = anthracyclines; ACEI = angiotensin-converting-enzyme inhibitors; ARB = angiotensin-receptor blockers; T = trastuzumab.

**Table 2:** Baseline echocardiographic characteristics in the studied population.

Parameter	CTRCD+ BP− *n* = 11 (A)	CTRCD+ BP+ *n* = 11 (B)	CTRCD−BP+ *n* = 11 (C)	CTRCD−BP− *n* = 11 (D)	P A−B	P A−C	P A−D	P B−C	P B−D	P C−D
LV Diastolic Dimension, mm	47.2 ± 4.3	47.5 ± 4.3	46.6 ± 3.4	49.7 ± 3.3	0.99	0.98	0.64	0.95	0.73	0.47
LV Mass Index, g/m^2^	79.4 ± 26.9	88.8 ± 17.8	85.5 ± 19.1	85.1 ± 21.9	0.82	0.95	0.96	0.99	0.98	0.99
Left Atrial Volume Index, ml/m^2^	27.3 ± 6.5	29.0 ± 9.9	28.8 ± 6.9	28.6 ± 4.4	0.96	0.98	0.98	0.99	0.99	0.99
E/A	1.04 ± 0.42	1.34 ± 0.42	1.06 ± 0.39	1.10 ± 0.34	0.45	0.99	0.99	0.51	0.66	0.99
e’ septal, cm/s	7.9 ± 1.4	9.0 ± 1.7	7.8 ± 1.7	7.7 ± 3.1	0.68	0.99	0.99	0.63	0.56	0.99
e’ lateral, cm/s	10.0 ± 1.8	11.4 ± 2.0	10.4 ± 2.9	9.7 ± 3.5	0.66	0.98	0.99	0.88	0.56	0.93
E/e’	7.7 ± 2.1	8.4 ± 1.4	7.9 ± 1.8	9.4 ± 3.6	0.93	0.99	0.55	0.96	0.86	0.62
LV Ejection Fraction, %	62.0 ± 3.6	64.0 ± 4.8	63.4 ± 4.4	62.5 ± 5.4	0.81	0.93	0.99	0.99	0.90	0.97
GLS, %	19.3 ± 3.0	20.6 ± 2.0	20.0 ± 2.6	19.2 ± 2.6	0.73	0.95	0.99	0.95	0.63	0.90
GWI, mmHg%	2076 ± 526	1846 ± 133	1846 ± 279	1834 ± 298	0.48	0.48	0.44	0.99	0.99	0.99
GCW, mmHg%	2405 ± 572	2146 ± 170	2168 ± 272	2205 ± 257	0.41	0.49	0.63	0.99	0.98	0.99
GWW, mmHg%	88 ± 65	90 ± 46	80 ± 45	96 ± 138	0.99	0.99	0.99	0.99	0.99	0.97
GWE	95.5 ± 3.0	94.6 ± 2.7	95.8 ± 1.7	95.5 ± 4.4	0.94	0.99	0.99	0.85	0.92	0.99

**Table 3:** Changes from baseline to follow-up in LV deformation, myocardial work, ejection fraction, and systolic blood pressure across the subsets.

Parameter	CTRCD+ BP− *n* = 11 (A)	CTRCD+ BP+ *n* = 11 (B)	CTRCD−BP+ *n* = 11 (C)	CTRCD−BP− *n* = 11 (D)	P A−B	P A−C	P A−D	P B−C	P B−D	P C−D
delta GLS, %	−3.40 ± 2.42	−5.71 ± 2.81	−1.05 ± 2.21	−1.01 ± 2.00	0.14	0.13	0.12	**<0.001**	**<0.001**	0.99
delta GWI, mmHg%	−493 ± 448	−110 ± 271	422 ± 292	−45 ± 281	**0.05**	**<0.001**	**0.01**	**0.004**	0.97	**0.01**
delta GCW, mmHg%	−541 ± 515	−114 ± 287	401 ± 233	−152 ± 284	**0.01**	**<0.001**	**0.03**	**0.008**	0.99	**0.005**
delta GWW, mmHg%	33.7 ± 63.8	60.8 ± 64.3	27.2 ± 46.2	−16.0 ± 102.2	0.85	0.99	0.46	0.75	0.12	0.58
delta GWE	−2.09 ± 3.38	−3.27 ± 3.49	−0.64 ± 1.91	−0.18 ± 2.78	0.83	0.72	0.52	0.24	0.13	0.98
delta EF, %	−11.6 ± 1.7	−14.4 ± 4.6	−0.2 ± 3.7	−0.6 ± 2.8	0.33	**<0.001**	**<0.001**	**<0.001**	**<0.001**	0.99
delta SBP, mmHg	−7.2 ± 18.4	25.7 ± 7.1	29.3 ± 5.2	−2.8 ± 6.0	**<0.001**	**<0.001**	0.67	0.88	**<0.001**	**<0.001**

Delta—follow-up value minus baseline value. The bold indicates *p*-values that are statistically significant.

## Data Availability

The data presented in this study are available on request from the corresponding author.
